# Virus Surveys in Olive Orchards in Greece Identify Olive Virus T, a Novel Member of the Genus *Tepovirus*

**DOI:** 10.3390/pathogens10050574

**Published:** 2021-05-08

**Authors:** Evanthia Xylogianni, Paolo Margaria, Dennis Knierim, Kyriaki Sareli, Stephan Winter, Elisavet K. Chatzivassiliou

**Affiliations:** 1 Plant Pathology Laboratory, Department of Crop Science, School of Agricultural Production, Infrastructure and Environment, Agricultural University of Athens, Iera Odos 75, Votanikos, 11855 Athens, Greece; eyaxylogian@gmail.com (E.X.); kiriakisareli@gmail.com (K.S.); 2 Leibniz-Institute DSMZ-German Collection of Microorganisms and Cell Cultures GmbH, 38124 Braunschweig, Germany; paolo.margaria@dsmz.de (P.M.); dek13@dsmz.de (D.K.); stephan.winter@dsmz.de (S.W.)

**Keywords:** phylogenetics, HTS, ArMV, CLRV, CMV, OLYaV, OlVT, SLRSV, TMV, TNV

## Abstract

Field surveys were conducted in Greek olive orchards from 2017 to 2020 to collect information on the sanitary status of the trees. Using a high-throughput sequencing approach, viral sequences were identified in total RNA extracts from several trees and assembled to reconstruct the complete genomes of two isolates of a new viral species of the genus *Tepovirus* (*Betaflexiviridae*), for which the name olive virus T (OlVT) is proposed. A reverse transcription–polymerase chain reaction assay was developed which detected OlVT in samples collected in olive growing regions in Central and Northern Greece, showing a virus prevalence of 4.4% in the olive trees screened. Sequences of amplified fragments from the movement–coat protein region of OlVT isolates varied from 75.64% to 99.35%. Three olive varieties (Koroneiki, Arbequina and Frantoio) were infected with OlVT via grafting to confirm a graft-transmissible agent, but virus infections remained latent. In addition, cucumber mosaic virus, olive leaf yellowing-associated virus and cherry leaf roll virus were identified.

## 1. Introduction

Olives (*Olea europaea* L.) have been cultivated in the Mediterranean area since prehistoric times. Greek olive cultivation ranks third in the EU, after Spain and Italy [1], and the olive agricultural and industrial sector is of immense importance for Greece, providing economic, social and environmental services. Olive trees are mainly propagated using semi-hardwood cuttings. This procedure has contributed over the years not only to the dissemination of the best olive materials, but also to the spread of pathogens, predominantly viruses. Fifteen viruses have been reported to naturally infect olive trees [2], of which olive latent virus 1 (OLV-1), olive mild mosaic virus (OMMV) (*Alphanecrovirus, Tombusviridae*), tobacco necrosis virus D (TNV-D) (*Betanecrovirus, Tombusviridae*), cucumber mosaic virus (CMV) (*Cucumovirus, Bromoviridae*), arabis mosaic virus (ArMV), cherry leaf roll virus (CLRV), olive latent ringspot virus (OLRSV) (*Nepovirus, Secoviridae*), tobacco mosaic virus (TMV) (*Tobamovirus, Virgaviridae*), olive latent virus 2 (OLV-2) (*Oleavirus, Bromoviridae*), olive latent virus-3 (OLV-3) (*Marafivirus, Tymoviridae*), strawberry latent ringspot virus (SLRSV) (*Secoviridae*) and olive vein yellowing associated virus (OVYaV) (*Closteroviridae*) are officially recognized [3]. In addition, olive yellow mottling and decline associated virus (OYMDaV), olive semilatent virus (OSLV) and olive leaf yellowing associated virus (OLYaV) have been proposed as distinct, but not yet approved, viral species [3]. In Greece, graft-transmissible, virus-like diseases of olive trees have been reported [4]; however, up-to-date information on the occurrence of viruses in Greek olive orchards is scarce. Only recently, SLRSV, CLRV, OLYaV and ArMV were detected in a germplasm collection of the Greek Institute of Olive Tree, Subtropical Plants and Viticulture (IOSV) in Chania (Crete) [5]. In earlier studies reporting from Greece, only OLV-3 was found to infect olive trees [6] with OLV-2 infecting *Ricinus communis* L. [7] and OMMV infecting spinach [8]. OLYaV was identified in Greek olive varieties grown in a Californian germplasm collection [9], although the origin of the virus remains unclear.

This study is the first nationwide survey to investigate the presence of viruses in Greek olive orchards. Visual inspections during the survey did not reveal trees with conspicuous symptoms indicating viral infection and thus confirmed the health status of the trees. To support the survey, a global analysis of olive transcriptomes was performed following a high-throughput sequencing (HTS) approach and subsequently a screening by RT–PCR. HTS permitted the discovery of a new viral species assigned to the genus *Tepovirus* (*Betaflexiviridae*) for which the name olive virus T (OlVT) is proposed. RT–PCR tests identified OlVT, CMV, CLRV and OLYaV infection in several olive trees.

## 2. Results

### 2.1. Complete Genome Sequence Determination of a Novel Tepovirus

**Figure 1:**
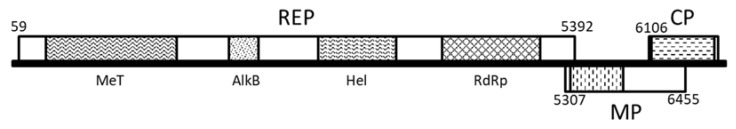
Schematic representation of the genomic organization of olive virus T isolate GR168. REP: replication-associated protein; MP: movement protein; CP: coat protein. Conserved motifs were predicted using Pfam (http://pfam.xfam.org/search/sequence (accessed on 18 March 2021): MeT, methyltransferase; AlkB, 2OG-FeII-Oxy2; Hel, helicase; RdRp, RNA-dependent RNA polymerase.

Analysis of the RNA-seq data from the pooled samples and BLAST alignments against a reference database revealed the presence of contigs with similarity to *Tepovirus* members, in particular to the prunus virus T (PrVT) isolate Aze239 [10] in pool MiV301 (Table S1). No other contigs with homology to viral sequences could be identified by BLASTn or BLASTp alignment. Screening of the RNAs from the samples composing the pool allowed us to trace an infected plant, corresponding to sample GR168, to its collection in Lamia, Fthiotida. Following standard RT–PCR and 5’-3’ RACE reactions, a complete genome of 6845 nt was assembled (isolate GR168). The genome showed an organization typical of members of the genus *Tepovirus* (Figure 1), containing three overlapping open reading frames (ORFs) that in direction 5’ to 3’ encode a replication-associated protein (REP, position 59–5395 nt; 1778 aa, 205.4 kDa), a putative movement protein (MP, position 5307–6458 nt; 383 aa, 42.6 kDa) and a capsid protein (CP, position 6106–6771 nt; 221 aa, 25.2 kDa). Two non-coding regions (UTR) of 58 nt and 74 nt in length are present at the 5’ and 3’ ends of the genome, respectively. As for other members of the *Betaflexiviridae* family, a polyA tail follows the 3’ UTR. Prediction of conserved domains revealed the presence of expected motifs in the viral proteins: methyltransferase (pfam01660, aa 43–334), 2OG-Fe(II) oxygenase (pfam13532, aa 673–767), viral RNA helicase (pfamPF01443, aa 959–1207), RNA-dependent RNA polymerase (pfam00978, aa 1355–1668) in the REP protein, the viral movement protein domain (pfam01107, aa 15–185) in the MP protein, and the tricho-coat domain (pfam05892, aa 9–210) in the CP protein. Analysis of sample GR170 (Table S1), collected in 2019 in the same orchard (Lamia, Fthiotida), allowed the assembly of the complete genome of a second OlVT isolate, GR170. The genome is 6846 nt in length and has three ORFs with identical sizes to the GR168 isolate, with a 5’ UTR of 58 nt and a 3’ UTR of 75 nt in length.

**Figure 2:**
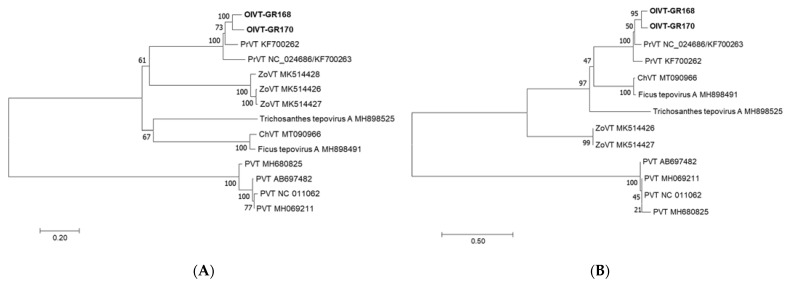
Neighbor-joining phylogenetic trees constructed on the deduced aa sequences of the replication-associated proteins (**A**) and capsid proteins (**B**) of accepted and putative members of the genus *Tepovirus*. The evolutionary distances are represented as the number of amino acid substitutions per site (PrVT: prunus virus T; ZoVT: zostera virus T; ChVT: cherry virus T; PVT: potato virus T).

Pairwise analysis at the whole genome level revealed 81.0% nt sequence identity between GR168 and GR170, and 74.5% and 74.8% nt identity, respectively, to the closest tepovirus, PrVT (KF700262). Alignment of the two OlVT isolates revealed 96.6% and 84.4% identity in the 5’ and 3’ untranslated regions (UTRs), respectively. At the ORF nt/aa level, the highest values were observed for the CP, with 87.8% and 91.4% identity at nt and aa level, respectively (Table S2). Phylogenetic analysis of the REP proteins of accepted and putative members of the genus *Tepovirus* (Figure 2A) showed that the olive isolates cluster in the clade with PrVT isolates, with the following closest clade composed of zostera virus T (ZoVT) isolates. Sequence identity at the aa level was 79.7–82.6% and 78.3–83.5% against PrVT accessions KF700263-KF700262 for GR168 and GR170 respectively. The CP-based tree (Figure 2B) showed the same clustering with PrVT, while the CP of ZoVT was more distant. Amino acid identity percentages were 84.6–86.9% and 85.5–87.3% aa against PrVT accessions KF700262-KF700263, for GR168 and GR170, respectively.

### 2.2. Transmission of OlVT by Grafting

OlVT was transmitted by grafting scions from GR168 infected olive field tree to Koroneiki, Arbequina and Frantoio varieties; however, they remained symptomless. Eight months after grafting, OlVT was detected by RT–PCR (Figure S1), and the sequences amplified from the grafted plants were identical to those of the originally grafted OlVT. The virus was not successfully transmitted to the Arbosana, Mastoidis, Mavrolia Messinias and Coratina varieties.

### 2.3. Molecular Variability of OlVT

**Figure 3:**
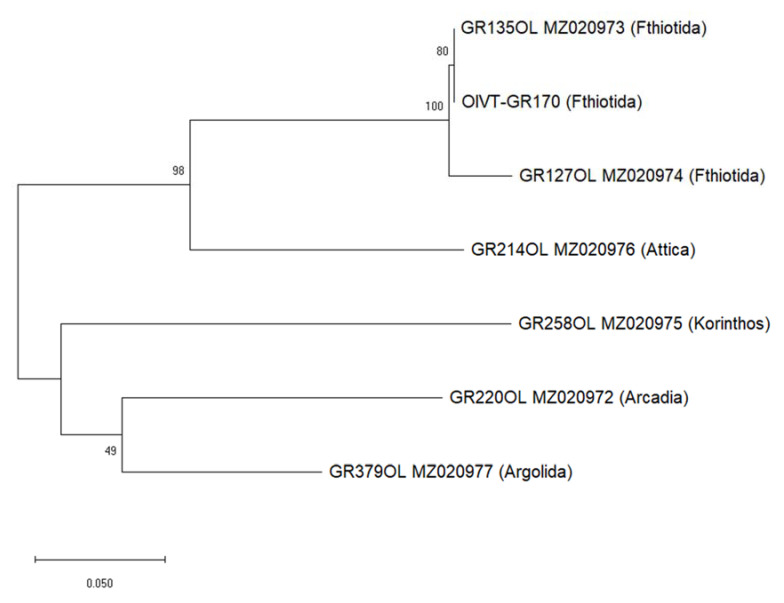
Maximum likelihood dendrogram depicting the phylogenetic relationships among the olive virus T’s (OlVT) partial movement protein and capsid protein sequences from the Greek isolates. Bootstrap values (%) analyzed by 1000 iterations are indicated on the corresponding branches.

In total, six sequences from six trees were obtained by the Sanger sequencing of RT–PCR products (GenBank accessions MΖ020972-MΖ020977), in addition to the sequence of isolate GR170, which was afterwards subjected to HTS. Nucleotide sequence similarity in the partial MP_CP region was in the range of 75.6–99.3%, with the highest diversity between the isolates obtained in two successive years from the same tree in Fthiotida (GR135OL and GR170) and the OlVT isolate from Korinthos (Peloponnese) (GR258OL) (Figure 3).

### 2.4. Viruses in Greek Olive Orchards

**Figure 4:**
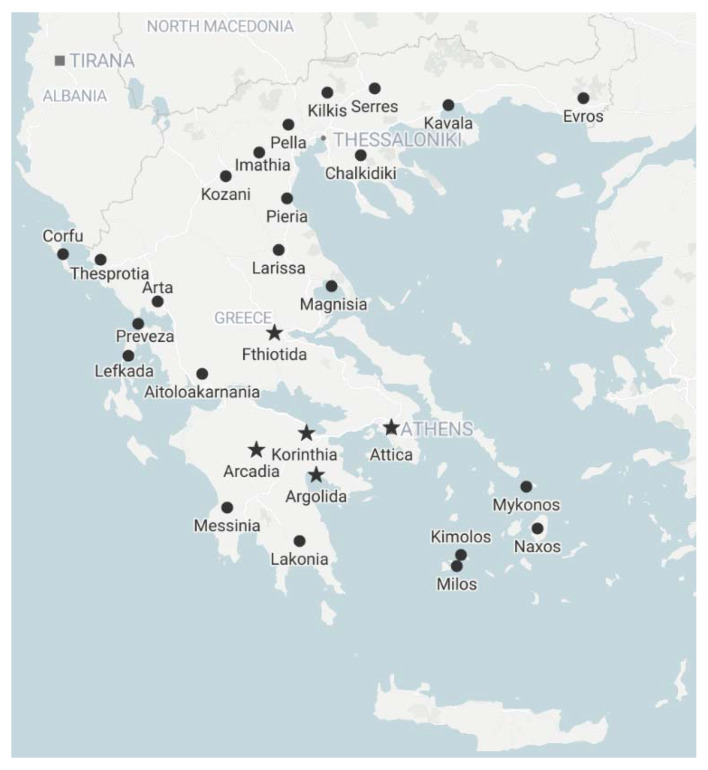
Map of Greece showing sampling areas (black dots) with olive orchards. Asterisks mark locations with OlVT-infected trees.

Visual inspections of olive trees in Greek orchards did not reveal conspicuous symptoms indicating viral infection. Viral testing by specific RT–PCR tests revealed viral infections in 13 out of the 158 samples (in total, 8.2% infection rate, Table S3). The novel OlVT was the most frequently detected virus in seven trees and five areas and was found in 4.4% of the tested trees. Besides Fthiotida (Lamia), OlVT was also detected in two distant fields in Argolida and one in each of Korinthia, Arkadia and Attica. Among the seven known olive viruses tested for, only three (CMV, CLRV, OLYaV) were detected: CMV was detected in five trees sampled in Agrinio, Attica, Argolida and Korinthia; OLYaV was detected in two trees, in Arkadia (Koutroufa) in the Peloponnese and Kilkis (Evropos) in Macedonia; and CLRV was detected in one tree in each of two areas (Megara and Paiania) in Attica (Figure 4).

### 2.5. Molecular Variability of Greek Isolates of Olive Viruses

#### 2.5.1. CMV RdRp Gene

Phylogenetic analysis of the RdRp gene showed that the five CMV isolates from olive trees (GenBank accessions MW805377-MW805381) shared a 91.55–100% sequence identity. One CMV isolate (GR195OL from Paiania, Attica) was grouped in subgroup IA of CMV; the isolates from Korinthia and Argolida in the Peloponnese (GR374OL, GR375OL and GR367OL) were identical and highly similar (99.16%) with that of GR45OL from Agrinio, and they were all clustered in CMV subgroup IB. The Greek olive isolates were 85.55–89.1% similar at the nt level, with the only three olive isolates available in the GenBank, from Albania (LR865402-LR865404).

#### 2.5.2. OLYaV HSP70h Gene

The amplification of the HSP70 homologue was successful in only one out of the two isolates of OLYaV (GR80OL) and resulted in a 339 nt sequence when excluding the primers (GenBank accession MW805382). This isolate showed higher sequence similarity (98.8%) with an isolate from the Ruget variety of French olive material kept in the USDA germplasm collection in California (HQ286488). Our isolate showed only 75.89% sequence similarity with the available Greek olive isolate (LR593885).

#### 2.5.3. CLRV 3’ UTR

Using the CLRV specific primers, we amplified an identical 341 bp long fragment from the 3’ UTR region for the two Greek olive isolates (GR186OL and GR252OL; GenBank accessions MW814913 and MW814914, respectively). These sequences showed only 91.9% similarity with two other identical Greek olive isolates already publicly available (MK936235, MK936236). Higher sequence similarity (93.84%) was observed with a *Plantago major* (KF779205) and an *Actinidia chinensis* (KF779202) isolate, both from New Zealand.

## 3. Discussion

Virus discovery studies in Greek olive orchards using HTS analysis enabled the assembly of the whole genome of a novel species of the genus *Tepovirus* [11] according to the species demarcation criteria in the *Betaflexiviridae* family [12]. In addition, graft transmission experiments unambiguously demonstrated its identity as a novel transmissible agent, for which the name olive virus T (OlVT) is proposed. To date, the genus *Tepovirus* includes potato virus T [11] and prunus virus T [3,10], while zostera virus T [13] and cherry virus T [14] have been recently suggested as new members.

Total RNA was used as the input for library preparation, rather than dsRNA or sRNAs, which can be used in alternative HTS approaches [15] and have also been successfully employed for virus discovery in woody hosts [10,14,16,17]. The total RNA strategy was selected to take advantage of the potential for the broad discovery of both DNA and RNA viruses offered by this approach and because of the *in-house* bioinformatics pipeline optimized for long reads. The number of raw reads obtained per sample was in the range of ca. 0.8 × 10^6^–2 × 10^6^ and was sufficient to generate contigs that covered most of the genome of the two assembled OlVT isolates, whose complete sequence was determined by the complementary Sanger sequencing of amplification products. Analysis of the assembled OlVT genomic sequences clearly indicated the presence of two distinct isolates of the same viral species, with an identity percentage of 81% at nt level, similar to that observed for the PrVT isolates previously described [10]. A similar variability was observed on the partial MP_CP sequences of all obtained OlVT field isolates.

The RT–PCR assay we developed allowed for the detection of OlVT in 4.4% of the tested trees (about half of those found to be infected by any virus), which were scattered in different areas in Central and Southern Greece. There are no known vectors for tepoviruses, and thus, the only known means of dissemination is infected vegetative propagation material. Nevertheless, similar to PVT [18], OlVT may be pollen- and seed-borne. In our graft transmission experiments, the important varieties Koroneiki, Arbequina and Frantoio became infected, but no symptoms were observed in the infected plants. The absence of symptoms is a common feature of most tepovirus infections and may facilitate OlVT spread via infected vegetative propagation material [2]. Other grafted varieties tested negative, which could be attributed to a lower viral load and/or sensitivity to OlVT; however, the number of plantlets tested was too low to safely conclude that. While graft transmission proved the infectivity of OlVT, additional studies are needed to record the sensitivity of further varieties and to assess any impacts of the viral infection.

The virus survey results indicate that the infection rate in Greek olive orchards is rather low (8.2%) compared to that in other countries [19,20,21,22,23,24], where incidences can reach 74.6% (e.g., in Tunisia) [24]. However, a high virus incidence, e.g., 93.8% in California [9] is often recorded from surveys in germplasm collections, which may not reflect field infection rates. In Greece, among 40 young (4–5 years old) plants in a germplasm collection of the IOSV in Chania, 62.5% were infected [5].

Besides OlVT, only CMV, OLYaV and CLRV were detected in olive trees, but at low prevalence. CMV is endemic in many economically important crops in Greece [25]. CMV in olive trees has been reported from a few countries, always in latent infections; a higher infection rate (24.7%) has been reported from Egypt [23,26,27]. Although most Greek CMV isolates are clustered in subgroup IA, most of the olive isolates fell into subgroup IB, which includes isolates of East Asian origin [28] as well as some host-adapted CMV isolates from Greek vegetables [25]. There were no satellite RNAs associated with CMV isolates from olive trees.

The olive-specific OLYaV, an unassigned species, possibly belonging to a new genus in the family *Closteroviridae* [29], was detected in two trees. Although this virus may be associated with a bright yellow discoloration in olive plants [2,26,30], changes in vegetative growth [31], or woody deformations [29], no such symptoms were observed. OLYaV appears to be one of the most widespread viruses in olives, with infection rates of up to 63% in Tunisia [2,24,26,32]. High OLYaV infection rates were also recorded in the germplasm repository of the USDA (National Clonal Germplasm Repository, Davis, California) where 93.8% of the tested varieties, including three Greek varieties, were found to be infected [9]. In Greece, 5% of the varieties kept in a germplasm collection of IOSV in Chania were infected with OLYaV [5]; however, with an isolate highly divergent from the one we collected.

CLRV was also detected in Greek olive trees. The virus is known to cause severe disease in several of its numerous woody hosts [33]. CLRV is reported in olives and walnuts in Greece [5,34,35]. The virus is reported in latent infection in olives from several countries with infection rates reaching 15% in Syria [19,26,27] and is transmitted via olive seeds at a rate of 41% [36], which may also contribute to viral spread. Several “host-specific” clusters of CLRV isolates have been suggested [37]; however, the CLRV isolate we collected appears to diverge from the Greek olive sequences already available [5]. In this study, the nepoviruses ArMV and SLRSV were not found, although they were recently detected in trees from a Greek germplasm collection [5].

From our olive virus survey, we can report the identification of a new virus species, OlVT, and the presence of a few known olive-infecting viruses in Greek orchards. None of the viral infections were associated with symptoms and the incidence was low. Contrasting the generally high virus incidence in “in situ” germplasm collections, the good health status of the Greek olive orchards can be stated. The fact that hardwood cuttings, the vegetative propagation materials, are the main means of viral spread, as well as the propagation of Greek autochthonous varieties by local nurseries and the, until recently, only very limited introduction of new materials from abroad may all be reasons for the low virus incidence. We found the new virus OlVT the most frequently, and this virus, which is not associated with symptoms, may have been present for quite some time in our country.

## 4. Materials and Methods

### 4.1. Plant Material

In spring 2017, 42 samples were collected from olive trees from different areas in Greece (Table S4), for virus discovery by HTS. In the following years, from 2018 to 2020, surveys for olive viruses were conducted in early June or late September. A total of 158 trees were sampled, specifically in Northern Greece (Kilkis (5: the number of tested trees), Pella (3), Evros (2), Kavala (2), Kozani (2), Serres (1) Chalkidiki (1)), Central Greece (Attica (38), Aitoloakarnania (4), Arta (5), Fthiotida (32), Magnisia (5)), Southern Greece (the Peloponnese peninsula; Argolida (16), Arkadia (2), Korinthia (16), Messinia (5), Lakonia (1)), as well as on some islands (Naxos (1), Milos (1), Kimolos (2), Mykonos (1), Corfu (10), Lefkada (3)) (Figure 4, Table S3). In order to assess the health status of the traditional Greek planting material, trees older than 20 years were considered for sampling. Because no conspicuous leaf symptoms could be observed, samples from up to 5 trees per field were randomly and individually collected. From each tree, two 1- to 2-year-old twigs of 20–30 cm in length were collected from each of the four quadrants of the canopy. Trees to be used as negative controls in our tests were obtained from tissue culture (Vitroplant Italia srl Soc. Agricola, Italy) and kept in an insect-proof greenhouse.

### 4.2. HTS and Genome Assembly

Total RNA was extracted from leaf tissues using an RNeasy Plant Mini Kit (QIAGEN, Germany), quality-checked by agarose gel electrophoresis and subsequently processed in the RNA-seq pipeline established at the DSMZ Plant Virus Department. The 42 samples collected in 2017 were processed in 11 pools (MiV300-310). An additional sample collected in 2019 that tested positive for OlVT in field surveys by RT–PCR was sequenced as a single (Table S4). Ribosomal RNA was depleted using a RiboMinus Plant Kit (Invitrogen) according to manufacturer´s instructions. Following cDNA and second-strand synthesis using random octamers, an Illumina library was prepared following the NexteraXT Library Kit protocol and sequenced on a MiSeq system, following a 2X301 paired-reads strategy. Bioinformatic analyses for contig assembly, BLAST alignment, genome reconstruction and annotation were performed in Geneious Prime version 2021.0.1. A custom plant virus database of RefSeq sequences downloaded from NCBI (generated on September 9th, 2020) was used as a reference for the local BLAST search. The complete genome sequences of the novel tepovirus were determined by the assembly of HTS contigs and Sanger-sequenced RT–PCR and 5’/3’ RACE products, obtained using primers designed on the partial genomic sequences in order to amplify PCR products spanning the gaps between the contigs or covering the terminal genomic regions (Table S5). The genome sequences of two OlVT isolates were submitted to GenBank under the accession number MW582811 (isolate GR168) and MW582812 (isolate GR170).

### 4.3. Detection of Olive Viruses by RT–PCR

Trees were individually tested for the presence of ArMV, CLRV, CMV, OLYaV, OlVT, SLRSV, TMV and TNV-D using RT–PCR tests, with the primers reported in Table S6.

Total RNA was extracted from 0.1 g of material from a mixture of cortical scrapings and leaves (including midrib) from each tree, which was powdered in liquid nitrogen prior to further processing using an RNeasy Mini Kit (QIAGEN, Germany). RNA was quantified in a NanoDrop 1000 Spectrophotometer (Thermo Fisher Scientific) and examined for integrity by agarose gels electrophoresis. The cDNA was synthetized from 1μg of total RNA using a RevertAid H Minus Reverse Transcriptase (Thermo Fisher Scientific) in the presence of 1.5 μg of random hexamers (Promega), in a 20µl reaction. Total RNAs extracted from freeze dried leaves infected with CMV, TMV, ArMV, TNV-D and SLRSV (DSMZ Plant Virus Collection), and OLYaV and CLRV RNA samples kindly provided by the Istituto per la Protezione Sostenibile delle Piante (Consiglio Nazionale delle Ricerche, Bari, Italy) were used as positive controls. For OlVT, a partial genome fragment cloned in a pDrive vector (QIAGEN, Germany) was used as positive control.

RT–PCR was carried out in a total volume of 50 μL, containing 1μL of cDNA template, 0.2 µM of each primer (Table S6), 0.2 mM dNTPs, MgCl_2_ so as to have a final concentration optimized for each primer set (2 mM for consNecro, 2.5 mM for SLRSV and 3 mM for OlVT, OLYaV, CMV, CLRV and TMV) and 1.25 units of DreamTaq DNA Polymerase, in 1X Buffer (Thermo Fisher Scientific). NoRT reactions and cDNA synthesized from healthy plants served as controls. PCR conditions were as follows: initial denaturation at 95 °C for 3 min, followed by 35 cycles of denaturation at 95 °C for 30 s, annealing for 30 s at the appropriate temperature for each primer set and extension at 72 °C for 1 min, with a final elongation step of 72 °C for 5 min. PCR products were separated in 1.5% agarose gels, stained with ethidium bromide, purified with a Nucleospin Gel extraction kit (Macherey Nagel), and Sanger-sequenced in both directions by a commercial sequencing service provider (Applied Biosystems).

### 4.4. Sequence and Phylogenetic Analyses

Pairwise similarities of OlVT with other tepoviruses were calculated according to the MUSCLE alignment on Sequence Demarcation Tool software (SDTv1.2) [38]. Phylogenetic trees on deduced amino acid sequences were generated in MEGA 7.0.25 [39] using the Neighbor-Joining method, with 500 bootstrap replicates, on a set of tepovirus sequences aligned using the online Clustal Omega tool (https://www.ebi.ac.uk/Tools/msa/clustalo/ (accessed on 18 March 2021).

The molecular variability of the other viral sequences was investigated by retrieving related sequences from GenBank using BLASTn (http://www.ncbi.nlm.nih.gov (accessed on 25 March 2021). Subsequently, sequences were aligned using MUSCLE with a percentage Identity Matrix (PIM) calculated using the SIAS tool (http://imed.med.ucm.es/Tools/sias.html (accessed on 25 March 2021)). MEGA version 7.0.26 [39] was used to infer evolutionary history using the Maximum Likelihood method, applying 1000 bootstrap iterations to validate the phylogenetic hypothesis of the Tamura-Nei model [40].

### 4.5. Transmission of the Novel Tepovirus by Grafting

Since OlVT is a novel virus for which no biological data exist, a confirmation of the transmissibility of the agent was provided by grafting. Scions of infected trees were obtained from shoots collected in late February 2020, from an orchard tree infected with OlVT isolate GR168 (Lamia, Fthiotida). Scions were cleft-grafted onto two or three plantlets each of Koroneiki, Arbequina, Arbosana, Mastoidis, Mavrolia Messinias (from Kostelenos nurseries, Greece), Frantoio and Coratina (from tissue culture; Vitroplant Italia srl Soc. Agricola, Italy). All recipient trees used as rootstocks were previously tested and found to be virus-free. Grafted plants were subsequently kept in insect-proof greenhouses at ambient temperatures ranging from 10 °C to 30 °C. Samples from the newly developing vegetation of the rootstock were taken after 3 months (in May) and 8 months (in October) and tested by RT–PCR for the presence of OlVT.

## Data Availability

All data generated or analyzed during this study are included in this published article (and its Supplementary Materials Files).

## Supplementary Materials

The following are available online at https://www.mdpi.com/article/10.3390/pathogens10050574/s1, Figure S1: Results of RT–PCR tests of olive plants grafted with olive virus T infected scions, Table S1: Overview of the HTS reads obtained from each sample library (raw, trimmed, normalized), viral contigs generated by de novo assembly, closest relative by BLAST analysis, and reads mapping the assembled final genome of the two olive virus T (OlVT) isolates (GR168 and GR170), Table S2: Percentage of identity of OlVT-GR170 and other (putative) tepovirus genomic sequences with OlVT-GR168, Table S3: Virus incidence in Greek olive orchards surveyed in 2018–2020 by RT–PCR, Table S4: Origin of the olive samples collected for virus discovery and composition of the pools used for HTS, Table S5: Primers designed on the partial olive virus T genomic sequences for determination of the whole genomic sequence by RT–PCR or RACE, Table S6: Primers used for the detection of olive viruses in Greek orchards by RT–PCR.

## References

[1] (2017). Eurostat. https://ec.europa.eu/eurostat/web/products-eurostat-news/-/DDN-20190301-1.

[2] Martelli G.P. (2013). A brief outline of infectious diseases of olive. Palestine Tech. Univ. Res. J..

[3] International Committee on Taxonomy of Viruses (ICTV). https://ictv.global/taxonomy/.

[4] Kyriakopoulou P.E. (1996). Olive fruit hump and olive fruit pox: Two new disease of possible viral origin. Phytopathol. Mediterr..

[5] Mathioudakis M.M., Saponari M., Hasiów-Jaroszewska B., Elbeaino T., Koubouris G. (2020). Detection of viruses in olive cultivars in Greece, using a rapid and effective RNA extraction method, for certification of virus-tested propagation material. Phytopathol. Mediterr..

[6] Alabdullah A., Elbeaino T., Minafra A., Digiaro M., Martelli G.P. (2009). Detection and variability of olive latent virus 3 in the mediterranean region. J. Plant Pathol..

[7] Grieco F., Parrella G., Vovlas C. (2002). An isolate of Olive latent virus 2 infecting castor bean in Greece. J. Plant Pathol..

[8] Gratsia M.E., Kyriakopoulou P.E., Voloudakis A.E., Fasseas C., Tzanetakis I.E. (2012). First report of Olive mild mosaic virus and Sowbane mosaic virus in spinach in Greece. Plant Dis..

[9] Al-Rwahnih M., Guo Y., Daubert S., Golino D., Rowhani A. (2011). Characterization of latent viral infection of olive trees in the national clonal germplasm repository in California. J. Plant Pathol..

[10] Marais A., Faure C., Mustafayev E., Barone M., Alioto D., Candresse T. (2015). Characterization by deep sequencing of prunus virus T, a novel *Tepovirus* infecting *Prunus* species. Phytopathology.

[11] Rubino L., Russo M., de Stradis A., Martelli G.P. (2012). *Tepovirus*, a novel genus in the family *Betaflexiviridae*. Arch. Virol..

[12] Adams M.J., Candresse T., Hammond J., Kreuze J.F., Martelli G.P., Namba S., Pearson M.N., Ryu K.H., Saldarelli P., Yoshikawa N., King A.M.Q., Adams M.J., Carstens E.B., Lefkowitz E.J. (2012). Family *Betaflexiviridae*. Virus Taxonomy. 9th Report of the ICTV.

[13] Goh C.J., Park D., Lee J.S., Davey P.A., Pernice M., Ralph P.J., Hahn Y. (2019). *Zostera* virus T-a novel virus of the genus *Tepovirus* identified in the eelgrass, *Zostera muelleri*. Acta Virol..

[14] Marais A., Šafářová D., Navrátil M., Faure C., Cornaggia D., Brans Y., Suchá J., Candresse T. (2020). Complete genome sequence of cherry virus T, a novel cherry-infecting tepovirus. Arch. Virol..

[15] Kutnjak D., Tamisier L., Adams I., Boonham N., Candresse T., Chiumenti M., De Jonghe K., Kreuze J.F., Lefebvre M., Silva G. (2021). A primer on the analysis of high-throughput sequencing data for detection of plant viruses. Microorganisms.

[16] Giampetruzzi A., Roumi V., Roberto R., Malossini U., Yoshikawa N., La Notte P., Terlizzi F., Credi R., Saldarelli P. (2012). A new grapevine virus discovered by deep sequencing of virus- and viroid-derived small RNAs in Cv Pinot gris. Virus Res..

[17] Maliogka V.I., Minafra A., Saldarelli P., Ruiz-García A.B., Glasa M., Katis N., Olmos A. (2018). Recent advances on detection and characterization of fruit tree viruses using high-throughput sequencing technologies. Viruses.

[18] Salazar L.F., Harrison B.D. (1978). Host range, purification and properties of potato virus T. Ann. Appl. Biol..

[19] Al Abdullah A., El Beaino T., Saponari M., Hallak H., Digiaro M. (2005). Preliminary evaluation of the status of olive-infecting viruses in Syria. EPPO Bull..

[20] Fadel C., Digiaro M., Choueiri E., Beaino T.E., Saponari M., Savino V., Martelli G.P. (2005). On the presence and distribution of olive viruses in Lebanon. EPPO Bull..

[21] Faggioli F., Ferretti L., Albanese G., Sciarroni R., Pasquini G., Lumia V., Barba M. (2005). Distribution of olive tree viruses in Italy as revealed by one-step RT-PCR. J. Plant Pathol..

[22] Varanda C., Cardoso J.M.S., do Rosário Félix M., Oliveira S., Clara M.I. (2010). Multiplex RT-PCR for detection and identification of three necroviruses that infect olive trees. Eur. J. Plant Pathol..

[23] Yousef S.A., Moawed S.M., El-Sayed M., Shalaby A.A. Detection of olive tree viruses in Egypt by one-step RT-PCR. Proceedings of the 21st International Conference on Virus and Other Graft Transmissible Diseases of Fruit Crops.

[24] Zellama M.S., Varanda C.M.R., Materatski P., Nabi N., Hafsa A.B., Saamali B.M., Chaouachi M., Félix M.R. (2019). An integrated approach for understanding the high infection rates of olive viruses in Tunisia. Eur. J. Plant Pathol..

[25] Valachas C.A., Giantsis I.A., Sareli K., Winter S., Zelezniakof E., Pentheroudaki Z., Chatzivassiliou E.K. (2021). Molecular analysis of Greek isolates of cucumber mosaic virus from vegetables show a low prevalence of satellite RNAs and suggest the presence of host-associated virus strains. Arch. Virol..

[26] Çağlayan K., Faggioli F., Barba M., Hadidi A., Barba M., Candresse T., Jelkmann W. (2011). Virus, phytoplasma and unknown diseases of olive trees. Virus and Viruslike Diseases of Pome and Stone Fruits.

[27] Afechtal M., Mounir M. (2020). Preliminary evaluation of the status of olive-infecting viruses in Morocco. Mor. J. Agric. Sci..

[28] Jacquemond M. (2012). Cucumber mosaic virus. Adv. Virus Res..

[29] Ruiz-García A.B., Candresse T., Canales C., Morán F., de Oliveira C.M., Bertolini E., Olmos A. (2020). Molecular characterization of the complete coding sequence of olive leaf yellowing-associated virus. Plants.

[30] Albanese G., Saponari M., Faggioli F., Muzzalupo I. (2012). Phytosanitary Certification. Olive Germplasm. The Olive Cultivation, Table Olive and Olive Oil Industry in Italy.

[31] Cutuli M., Lo Bianco R., Marra F.P., Caruso T. (2011). Vegetative growth and ecophysiological aspects in young olive plants inoculated with olive leaf yellowing associated virus (OLYaV). Acta Italus Hortus..

[32] Campos M.D., Zellama M.S., Varanda C., Materatski P., Peixe A., Chaouachi M., Félix M. (2019). do R. Establishment of a sensitive qPCR methodology for detection of the olive-infecting viruses in Portuguese and Tunisian orchards. Front. Plant Sci..

[33] Büttner C., von Bargen S., Bandte M., Myrta A., Hadidi A., Barba M., Candresse T., Jelkmann W. (2011). Cherry leaf roll virus. Virus and Virus-Like Diseases of Pome and Stone Fruits.

[34] Kaponi M.S., Kyriakopoulou P.E. (2008). Olive viruses in Greece. Phytopathol. Mediterr..

[35] Sclavounos A.P., Kyriakopoulou P.E., Holeva M.C., Kostas P., Voloudakis A.E. (2008). Detection of Cherry leaf roll (CLRV) from walnut (*Juglans regia* L.) in Greece. Phytopathol. Mediterr..

[36] Saponari M., Savino V., Martelli G.P. (2002). Seed transmission in olive of two olive infecting viruses. J. Plant Pathol..

[37] Rebenstorf K., Candresse T., Dulucq M.J., Büttner C., Obermeier C. (2006). Host species-dependent population structure of a pollen-borne plant virus, cherry leaf roll virus. J. Virol..

[38] Muhire B.M., Varsani A., Martin D.P. (2014). SDT: A virus classification tool based on pairwise sequence alignment and identity calculation. PLoS ONE.

[39] Kumar S., Stecher G., Tamura K. (2016). MEGA7: Molecular evolutionary genetics analysis version 7.0 for bigger datasets. Mol. Biol. Evol..

[40] Tamura K., Stecher G., Peterson D., Filipski A., Kumar S. (2013). MEGA6: Molecular evolutionary genetics analysis version 6.0. Mol. Biol. Evol..

