# Supplementary material for: Virus Surveys in Olive Orchards in Greece Identify Olive Virus T, a Novel Member of the Genus Tepovirus
Source: Pathogens. 2021 May 8;10(5):574. doi: 10.3390/pathogens10050574 (PMC8150953; doi:10.3390/pathogens10050574)
Supplement: Supplementary file 1 [file pathogens-10-00574-s001.zip › Supplementary Figures and Tables/FigureS1.pptx]

## Slide 1
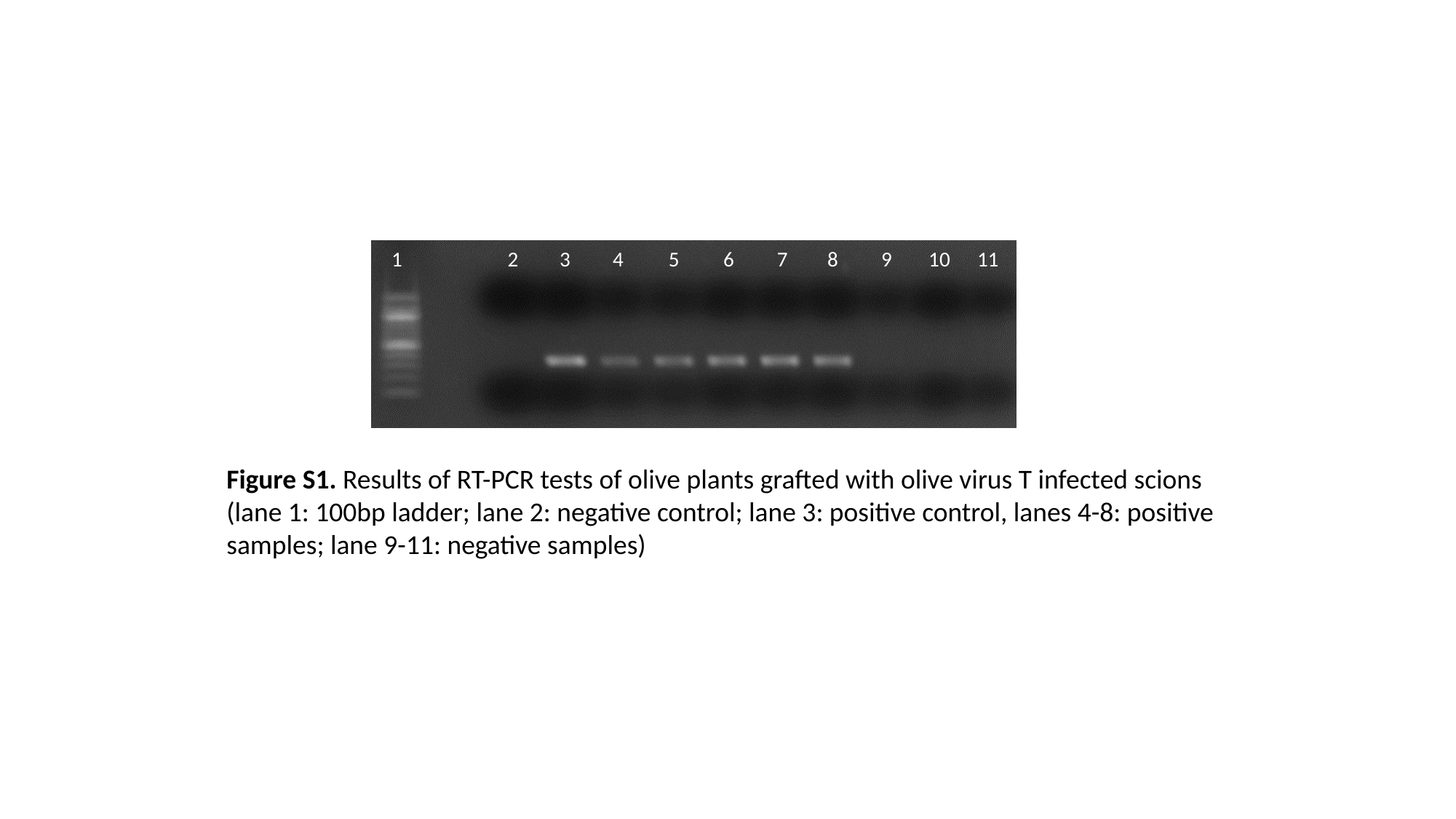

8
7
6
4
5
11
10
9
3
2
1
Figure S1. Results of RT-PCR tests of olive plants grafted with olive virus T infected scions (lane 1: 100bp ladder; lane 2: negative control; lane 3: positive control, lanes 4-8: positive samples; lane 9-11: negative samples)
